# The complex formed between a synthetic RNA aptamer and the transcription repressor TetR is a structural and functional twin of the operator DNA–TetR regulator complex

**DOI:** 10.1093/nar/gkaa083

**Published:** 2020-02-13

**Authors:** Florian C Grau, Jeannine Jaeger, Florian Groher, Beatrix Suess, Yves A Muller

**Affiliations:** 1 Lehrstuhl für Biotechnik, Department of Biology, Friedrich-Alexander University Erlangen-Nuremberg, Henkestr. 91, D-91052 Erlangen, Germany; 2 Department of Biology, Technische Universität Darmstadt, Schnittspahnstrasse 10, D-64287 Darmstadt, Germany; 3 Centre for Synthetic Biology, Technische Universität Darmstadt

## Abstract

RNAs play major roles in the regulation of gene expression. Hence, designer RNA molecules are increasingly explored as regulatory switches in synthetic biology. Among these, the TetR-binding RNA aptamer was selected by its ability to compete with operator DNA for binding to the bacterial repressor TetR. A fortuitous finding was that induction of TetR by tetracycline abolishes both RNA aptamer and operator DNA binding in TetR. This enabled numerous applications exploiting both the specificity of the RNA aptamer and the efficient gene repressor properties of TetR. Here, we present the crystal structure of the TetR-RNA aptamer complex at 2.7 Å resolution together with a comprehensive characterization of the TetR–RNA aptamer *versus* TetR–operator DNA interaction using site-directed mutagenesis, size exclusion chromatography, electrophoretic mobility shift assays and isothermal titration calorimetry. The fold of the RNA aptamer bears no resemblance to regular B-DNA, and neither does the thermodynamic characterization of the complex formation reaction. Nevertheless, the functional aptamer-binding epitope of TetR is fully contained within its DNA-binding epitope. In the RNA aptamer complex, TetR adopts the well-characterized DNA-binding-competent conformation of TetR, thus revealing how the synthetic TetR-binding aptamer strikes the chords of the bimodal allosteric behaviour of TetR to function as a synthetic regulator.

## INTRODUCTION

Short and long non-coding RNAs are important regulators of gene expression in all kingdoms of life. Consequently, RNA molecules have become prominent in synthetic biology, and small regulatory RNAs, synthetic riboswitches and allosterically controlled ribozymes are being investigated as regulatory devices in the design of genetic circuits and networks (1). Rapid progress in this development resulted in a swift transition from simple proof of concept to sophisticated applications targeting complex problems (2,3).

Synthetic RNA devices are unique due to their modular nature that allows the simple and straightforward linkage of different domains, e.g. between a sensor and an actuator. Thus, a whole range of different functions may be united in one RNA molecule or incorporated into a mRNA. At the same time, RNA-based sensor domains that bind their target with extraordinary high affinity and specificity can be identified *de novo* by *in vitro* selection (SELEX, Systematic Evolution of Ligands by Exponential enrichment) (4,5). These so called aptamers can adopt defined three-dimensional structures such as binding pockets or cleft-like interaction surfaces similar to those found in antibodies (6–9). One approach is to develop aptamers that target proteins involved in regulatory mechanisms such as, for example, bacterial repressor proteins. One interesting example is an RNA aptamer that is able to block operator binding in the bacterial transcription regulator TetR (10).

The TetR family of bacterial repressors is one of the largest families of transcriptional regulators (11). Eponymous TetR is a homodimeric α-helical protein, and each monomer comprises 10 α-helices (12,13). The tertiary structure of TetR family members consists of two domains: an N-terminal nucleotide-binding domain (NBD) and a C-terminal effector-binding domain (EBD) that also contains the dimerization interface. In the absence of its natural ligand tc, TetR binds to the DNA *tetO* operator sequence and thereby represses downstream genes. Upon tc binding to the EBD, TetR undergoes an allosteric rearrangement that increases the separation of the NBDs, abolishes *tetO* operator binding and alleviates gene repression (14,15). TetR repression is highly specific and extremely sensitive, and these properties, along with the favorable pharmacokinetics of tc and its derivatives, have made the so-called Tet-system an ideal tool for gene regulation in both prokaryotic and eukaryotic cells (16,17).

The TetR-binding RNA aptamer was identified by a combination of *in vitro* selection for TetR binding and an *in vivo* screening for aptamer activity *via* a transcription reporter assay (10). The identified aptamer was able to displace TetR from *tetO in vivo*, thus representing an alternative RNA-based activator of TetR-controlled transcription (10,18). Although no selection pressure was applied to this end, the allosteric rearrangement induced in TetR upon tc binding also compromises aptamer binding, consequently putting the dissociation of the TetR-RNA aptamer complex under the control of tc (18).

**Figure 1.**
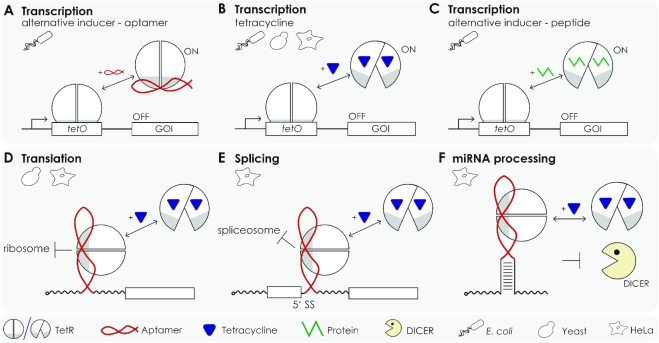
The TetR aptamer as versatile genetic device. (**A**) The TetR aptamer can activate TetR controlled gene expression by interfering with the DNA operator binding of the Tet repressor (10). Thus, it represents an alternative inducer to (**B**) tetracycline- or (**C**) peptide-mediated induction of TetR (26). (**D**) The aptamer also can be exploited to reversibly and efficiently control translation (24), (**E**) mRNA splicing (24) or (**F**) miRNA processing (22).

With the advent of the TetR-binding aptamer, the multitude of devices that make use of the stringent repressor properties of TetR has significantly expanded (Figure 1). While the original publication focused on the control of gene expression in *Escherichia coli* (10), portability and broader applicability of the system was documented with its successful use in the protozoon *Plasmodium falciparum* and in yeast (19,20). The addition of an additional regulatory layer to the TetR aptamer system was the design of a theophylline responsive TetR aptamer (a theophylline-aptamer fused to a TetR aptamer) proven to be functional (21). Later, the TetR aptamer was applied to control miRNA biogenesis in human cells (22). In this specific approach, the TetR aptamer replaces the natural terminal loop of precursor miRNAs, which, upon binding of TetR, leads to the inhibition of miRNA processing by Dicer *via* steric hindrance (23). The inhibition is fully reversible after addition of doxycycline (dox), thus providing a system that allows control over intracellular miRNA levels and, consequently, their gene-silencing properties (22). The very recent approach exploits the TetR-binding aptamer for the control of translation and pre-mRNA splicing. For this, the TetR aptamer was placed either in the 5′UTR or near the 5′SS in such a way that it interferes with initial steps of translation or splicing, respectively, when bound by TetR. Repression is fully relieved by the addition of dox that leads to the release of TetR from the RNA. Regulation was demonstrated for a multitude of different introns and target genes (24).

Despite the considerable body of research that yielded many interesting innovative applications for the TetR-binding aptamer, no structural information on the TetR–aptamer complex has yet been reported. Here, we present the crystal structure of the full-length TetR protein in complex with the TetR-binding aptamer. Our study provides atomic insight into the protein-RNA interface by combining X-ray crystallography, mutational analyses and biophysical assays. Our mechanistic studies show in detail how the TetR-binding aptamer uses the entire gamut of TetR allostery to exert its function.

## MATERIALS AND METHODS

### RNA aptamer, dsDNA and protein production and purification

Two variants K1 and K2 of the TetR-binding RNA aptamer were produced. They differ in the length and base composition of the two stem regions P1 and P2 and the apical loop (Supplementary Figure S1). K1 and K2 were *in vitro* transcribed by run-off transcription from an EcoRI-linearized pSP64 plasmid using a T7 promoter and purified according to a previously established protocol (18). For precise 3′ ends, the primary transcripts contained self-cleaving hammerhead ribozymes (all plasmid sequences are available upon request).

Complementary DNA oligonucleotides containing the native 13 bp-long *tetO* operator sequence were purchased from Eurofins Genomics (Ebersberg) and hybridized by cooling from 368 to 293 K in ITC buffer (20 mM potassium phosphate pH 7.4, 50 mM NaCl) to form double-stranded DNA (dsDNA, Supplementary Table S1) (25). The TetR protein used in all experiments is of type BD’. This is a cysteine-free, chimeric protein that comprises residues 1–187 from TetR class B and residues 188–208 from TetR class D (TetR (B1–B187:D188–D208; C68S, C88N, C121T and C144S)). Protein production and purification were achieved as reported earlier (26,27).

### Protein-RNA complex formation, purification and co-crystallization

The TetR–aptamer complex was prepared by mixing a solution containing 6 mg/ml purified TetR with the TetR-binding aptamer at a molar ratio of 1:1.15 in 20 mM Tris–HCl, pH 8.0, 150 mM NaCl and 5 mM MgCl_2_ and incubating the mixture for 1 h on ice. Next, a preparative gel filtration run was performed, using a Superdex 200 10/300 GL column (GE Healthcare, Munich, Germany) with the same buffer, to remove any excess of unbound aptamer from the TetR–RNA complex. TetR in complex with either aptamer K1 or K2 was concentrated to protein concentrations of 7 mg/ml and 16 mg/ml, respectively, as determined *via* Bradford assay (28). Single crystals of the TetR–aptamer K1 complex could be obtained after 4 months *via* the sitting-drop vapor diffusion method in 96-well plates (drop volume 0.2 μl, protein to reservoir solution ration of 1:1) at 20°C using the following condition: 4% (v/v) Tacsimate pH 6.0, 12% (w/v) PEG 3350. The crystals were flash frozen in liquid nitrogen using 20% (v/v) ethylene glycol as cryoprotectant for data collection at 100 K. Single crystals of the TetR–aptamer K2 complex could be obtained after 14 days *via* the sitting-drop vapor diffusion method in 96-well plates (drop volume 0.3 μl, protein to reservoir solution ration of 2:1) at 20°C using the following condition: 15% (*v/v*) pentaerythritol propoxylate (5/4 PO/OH), 0.2 M NaCl, pH 6.0, 0.1 M MES–NaOH. The crystals were flash frozen in liquid nitrogen without the use of any cryoprotectant.

### Diffraction data collection, structure determination and refinement

Diffraction data sets of TetR-RNA aptamer K1 and TetR–RNA aptamer K2 complex crystals were collected from single crystals at synchrotron beamline BL14.2 at BESSY II in Berlin to resolutions of 2.7 and 2.9 Å, respectively (29). Data were indexed and integrated using program XDS and scaled with XSCALE (30). Initial phases were obtained *via* molecular replacement with program PHASER using the apo-structure of TetR type BD’ (PDB code: 2NS7) as a search model (31). Several short fragments of double- and single-stranded RNA were iteratively added to the initial model in subsequent PHASER runs. The models were completed *via* alternating cycles of manual building in COOT and automated refinement with PHENIX (32,33). The quality of the final model was validated with MolProbity (34). All structural illustrations were prepared with Chimera (35).

### Analytical size exclusion chromatography

Analytical size exclusion chromatography (SEC) was performed in a 20 mM Tris–HCl, pH 8.0, 150 mM NaCl, 5 mM MgCl_2_ buffer on a Superdex 200 5/150 GL column (GE Healthcare). The macromolecules were investigated at concentrations of 50 μM (with regard to dimeric TetR, dsDNA and single-stranded RNA aptamer) and mixed at equimolar ratios.

### Electrophoretic mobility shift assay

*In vitro* transcribed RNA aptamer K1 was dephosphorylated with calf intestinal phosphatase (Roche, Mannheim) for 1 h at 37°C. Dephosphorylated RNA (10 fmol) was 5-labeled with γ-[^32^P]-ATP in 20 μl volume using polynucleotide kinase for 1 h at 37°C. The reaction was stopped with 2× loading buffer containing 7 M urea. The RNA was purified using preparative 10% polyacrylamide gels containing 7 M urea. The signals were detected using autoradiography. The RNA was extracted from the gel, eluted using 300 mM sodium acetate for 1 h, ethanol-precipitated and resuspended in H_2_O.

5′-[^32^P]-labeled aptamer RNA was incubated with increasing amounts of TetR variants (0–1000 nM) for 30 min at room temperature in 20 μl volumes in 50 mM Tris–HCl (pH 8.0), 10 mM MgCl_2_, 25 mM NaCl and 25 μg/ml yeast tRNA and subsequently loaded onto a 10% native PAA gel using 1× TB buffer (0.89 M Tris, 0.89 M boric acid, pH 8.3). Complex formation was resolved on 10% polyacrylamide gels as described above. To obtain *K*_A_ values expected in the low nanomolar range, the assay was performed with RNA concentrations equivalent to 50 000 cpm. The relative intensities of the band corresponding to the free RNA and RNA–protein complex at different protein concentrations were determined by phosphoimaging.

### Isothermal titration calorimetry

Isothermal titration calorimetry (ITC) measurements were performed in a Nano ITC standard cell (TA Instruments, New Castle, USA) at 25°C at a constant stirring rate of 300 rpm. Proteins and aptamer were dialyzed extensively against ITC buffer (20 mM KH_2_PO_4_/K_2_HPO_4_ pH 7.4, 50 mM NaCl) prior to any titration experiment. DNA oligonucleotides were hybridized and diluted to working concentrations in ITC buffer. All samples were degassed for 15 min at 900 rpm under vacuum using a degassing station (TA Instruments) prior to the titrations. Titration of either TetR or variant TetR-Q38A to the aptamer was performed with a 175 μM protein (dimeric TetR or TetR-Q38A) and a 15 μM aptamer concentration. Titration of variant TetR-Y42A to the aptamer was performed with 345 μM dimeric TetR-Y42A and 15 μM aptamer. Each experiment consisted of 35 × 5 μl injections with 360 s-long pauses in-between injections. The first injection volume was set to 2 μl to remove mixed reactants in the needle tip resulting from diffusion during the equilibration period of the instrument. A blank titration of the respective TetR variant into ITC buffer was used to account for any heat resulting from mixing and dilution. Data were analyzed using the NanoAnalyze software version 3.7.5 (TA Instruments).

### Structure comparisons

A number of r.m.s.d. values were calculated to compare the orientations of the NBDs and EBDs of TetR in the TetR-RNA aptamer complex with those in previous TetR structures. The structures were superimposed first using the Cα-positions of a set of conformationally invariant residues from the two EBDs present in dimeric TetR (27). These consisted of the following segments: 48–66, 73–103, 108–129, 131–137, 139–151, 166–173, 179–180 and 183–202 and are present twice in dimeric TetR. Following the superposition of the EBDs, the r.m.s.d. values between the NBDs in the different complexes were calculated (for Cα-positions, only) without further optimization of the structural alignment. Additionally, inter-residue distances between identical residues present in the compared complexes were measured.

## RESULTS

### Crystal structure of the TetR–RNA aptamer complex

**Figure 2.**
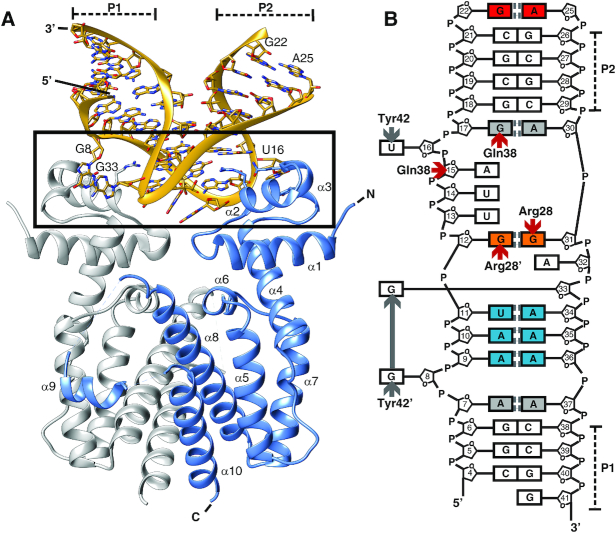
Structure of the TetR-aptamer complex. (**A**) Cartoon representation of dimeric TetR (in blue and gray) in complex with the RNA aptamer K1. The N-terminal NBDs of TetR are formed by helices α1 through α3 and the EBDs by helices α4 through α9. The TetR-RNA aptamer interface is framed. (**B**) Schematic summary of the base pairings within the aptamer as well as of the side chain-specific TetR interactions. Nucleobases in canonical Watson-Crick base pairings are colored white, *trans* Watson-Crick/Hoogsteen pairings blue and *trans* sugar-edge/sugar-edge pairings orange. The two non-canonical Watson–Crick/Watson–Crick pairings are shown in gray and the sugar edge/Hoogsteen pair in red. Hydrogen bond interactions between TetR and RNA aptamer are indicated by red arrows and selected π–π-stacking interactions by gray arrows. The two stems P1 and P2 are labeled.

The crystal structure of TetR in complex with the 43-nucleotide-long TetR-binding RNA aptamer K1 was determined at 2.7 Å resolution (Table 1). The complex consists of one TetR dimer bound to a single aptamer (Figure 2A). The conformations of the two protein chains present in dimeric TetR are highly similar. The two chains are related by a two-fold symmetry axis and deviate from each other by an r.m.s.d. value as low as 0.38 Å (calculated using C_α_-atoms). Helix α9 of the all-helical TetR molecule is only partially resolved in one TetR chain and missing in the second chain. In addition, electron density is missing in both protomers for the loops connecting helix α9 to the adjacent helices α8 and α10. In the complex, TetR adopts a conformation that is highly similar to the conformation that TetR adopts for DNA binding and which has been extensively characterized before (27).

**Table 1.** Crystallographic data collection and refinement statistics of TetR in complex with the RNA aptamer K1

	TetR in complex with aptamer K1
**Protein data bank** **accession number**	6SY4
**Data collection**	
X-ray source	Beamline HZB Berlin MX 14.2
Wavelength (Å)	0.9184
Space group	*P* 4_2_2_1_2
Cell dimensions	
*a, b, c* (Å)	96.03, 96.03, 163.01
*α, β, γ* (°)	90, 90, 90
Resolution range (Å)	15.8–2.7 (2.8–2.7)^a^
*R_meas_* (%)	20.5 (353.9)
*CC_1/2_*	0.999 (0.345)
CC*	1.000 (0.716)
*I/σI*	17.6 (1.0)
Completeness (%)	99.3 (100.0)
Redundancy	26.1 (25.2)
Wilson B-Factor (Å^2^)	75.3
**Refinement**	
Resolution (Å)	15.7–2.7
No. reflections	21641 (2123)
*R*_work_/*R*_free_ (%)	20.1/24.6
*CC_work_*	0.961 (0.615)
*CC_free_*	0.933 (0.546)
No. atoms	
Macromolecules	3699
Solvent	19
*B*-factors (Å^2^)	
Mean	80.4
Macromolecules	80.5
Solvent	64.5
R.m.s. deviations	
Bond lengths (Å)	0.004
Bond angles (°)	0.800
Ramachandran plot	
Favored (%)	98.6
Allowed (%)	1.2
Outliers (%)	0.0
Molprobity Clashscore	3.6

^a^The values for the highest resolution shell are reported in parentheses.

The aptamer adapts a hairpin-like L-shaped structure in the complex. The two arms of the L-shape consist of a 3 bp-long closing stem P1 and a 4 bp-long P2 stem. Both stems form A-form helices (Figure 2, Supplementary Figure S1). The first helix is flanked by one non-canonical base pairing (base pair A7:A37), and the second helix by two, i.e. one at the beginning of the P2 stem (base pair G17:A30) and one at the end (G22:A25). Whereas A7:A37 classifies as an A-A *trans* Watson-Crick/Watson-Crick base pair, G17:A30 classifies as a G–A *cis* Watson–Crick/Watson–Crick base pair and the terminal bases G22 and A25 form a G–A sugar edge/Hoogsteen pair (36). Although in the complexed aptamer, the P2 stem is closed-off by an apical GAAA loop on one side, no density is visible for the central two nucleotides of the tetraloop. Therefore, these nucleotides were omitted in the final model (Supplementary Figure S1). Overall, 36 nucleotides, i.e. nucleotides 4–22 and 25 to 41, of the 43-nucleotide-long aptamer K1 could be modeled with confidence.

Seventeen nucleotides form the middle section of the aptamer. In this section, four non-canonical base pairs occur. Base pairings A9:A36 and A10:A35 qualify as *trans* A–A Watson–Crick/Hoogsteen pairs, the U11:A34 pairing is a *trans* A–U Watson–Crick/Hoogsteen pair and G12:G31 is a G-G *trans* sugar-edge/sugar-edge base pair (36).

Bases are stacked continuously in C4–A7 and A9–G12. Nucleobase A7 stacks with G6 and A9, whereas G8 is flipped outwards and points towards the protein with torsion angles adopting non-standard values (ζ = –111.0°; α = 53.1°; β = –154.4°; γ = 56.8°). Nucleobase A15 (ζ = –84.3°; α = 78.0°; β = –162.0°; γ = –178.3°), which precedes the flipped-out U16 (ζ = 48.5°; α = –98.3°; β = –122.9°; γ = 136.7°), also fails to stack with the preceding nucleobase U14. Bases G17–G22 are again stacked continuously, as are A25–G31. A32 (ζ = 87.3°; α = –69.9°; β = 166.3°; γ = 59.4°), which precedes the flipped-out G33 (ζ = –170.4°; α = 51.3°; β = 179.4°; γ = 43.4°), is not involved in regular stacking interactions with adjacent nucleobases. In contrast, bases A34–G41 are again stacked continuously. All nucleotides of the RNA aptamer adopt an anti-conformation.

The L-shaped conformation of the aptamer and the presence of two A-form helices in the two stems of the L-shaped aptamer suggest that the two-fold rotational symmetry that relates the TetR chains in dimeric TetR possibly extends to the structure of the aptamer and forms a pseudo 2-fold symmetry axis in the complex. However, any attempts to superimpose the RNA aptamer onto itself while rotating the TetR–RNA aptamer complex around the dyad axis of the TetR homodimer resulted in a poor overall match (Supplementary Figure S2). Nevertheless, the phosphorous atoms of nucleotides A15 and G33 become positioned within distances of only 0.4 and 0.9 Å of phosphorous atoms of their respective counterparts, G33′ and A15′, following a 180° rotation around the dyad axis. At the same time, this structural correspondence increasingly diverges towards the ends of the aptamer (Supplementary Figure S2B). This clearly shows that the two-fold symmetry characterizing dimeric TetR does not apply in the form of a pseudo two-fold symmetry to the entire complex. Moreover, this finding is significantly different from the previously characterized TetR–DNA complex. In the latter, the palindromic nature of the *tetO* DNA sequence allows for the two-fold symmetry axis of the TetR dimer to extend to the DNA segment, so that the entire TetR-DNA complex displays a two-fold symmetry (13).

As mentioned above, the apical GAAA tetraloop (nucleotides 22–25) could only be modelled in part. A crystal packing analysis suggested that no space is available for the placement of the central AA nucleotides. It is therefore possible that these nucleotides are absent in the crystal, most likely due to a random proteolytic degradation event. This would also explain the 4-month incubation time required for crystal growth. To further investigate this, a second TetR–aptamer complex was crystallized. The crystal structure of TetR in complex with a shortened 39-nucleotide-long TetR-binding RNA aptamer variant K2 was determined at 2.9 Å resolution (Supplementary Figure S3A, Supplementary Table S2). In this complex, nucleotides 2–37 could be modelled continuously, including the apical UUCG tetraloop of which the tip was missing in the previous structure. K2 shared 74% sequence identity with K1, and the overall fold of aptamer K2 was identical to that of aptamer K1 (Supplementary Figure S3B). Apart from the fully formed apical tetraloop, the only two additional differences were a shortened 3 bp-long stem P1 and a likewise altered stem P2 (Supplementary Figure S1). Since all interactions between TetR and aptamer were identical in the K1 and K2 complexes and owing to the higher resolution of the TetR–RNA aptamer K1 complex, we limit ourselves to the description of the latter and refer to the K1 complex as the TetR–RNA aptamer complex from here on.

### The TetR–RNA interface

A direct consequence of the symmetry disparity between dimeric TetR and the monomeric aptamer is that the two NBDs of TetR interact with two different aptamer surface patches. The contact surface between aptamer and TetR is discontinuous, and the two TetR–NBDs contribute 420 and 430 Å^2^ to the binding epitope (850 Å^2^, in total). Thirteen residues from dimeric TetR (seven residues from the first protein chain and six residues from the second) are located within 3.7 Å of any aptamer residues. Interatomic contacts involve hydrogen bonding, van der Waals, π–π-stacking and cation–π interactions. Examples for sequence-unspecific contacts are two single hydrogen bonds formed between the phosphate group of either nucleotide A15 or G33 and the backbone atoms of Lys48 (in case of A15) and Lys48′ (G33) of TetR (where a prime denotes residues from the second TetR monomer chain). An additional, aptamer-unspecific hydrogen bond is formed between the side chain of Gln38 and the 2′-hydroxyl group of the nucleotide A15.

**Figure 3.**
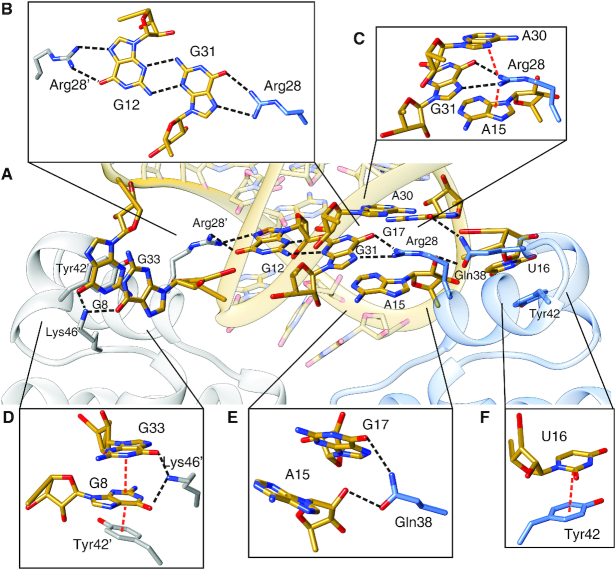
Atomic details of the interaction between TetR and RNA aptamer. (**A**) Close-up view of the TetR-aptamer interface. (**B**) Stick representation of the *trans* sugar-edge/sugar-edge base pairing between nucleobases G12 and G31 (in yellow) and interacting amino acids Arg28 (blue) and Arg28′ (gray). (**C**) Interaction between Arg28 (blue) and its interaction partners: A15, A30 and G31, (**D**) between Tyr42′, Lys46′ (gray), G8 and G33, (**E**) between Gln38 (blue), A15 and G17 and (**F**) between Tyr42 (blue) and U16. In all panels, dotted lines indicate hydrogen bonds (black) and cation–π or π–π-stacking interactions (red).

Six TetR residues, i.e. Arg28, Arg28′, Gln38, Tyr42, Tyr42′ and Lys46, are involved in sequence-specific interactions with the aptamer (Figures 2B and 3). Arg28 and Arg28′ each form a bidental hydrogen-bonding interaction with a guanine nucleobase, specifically G31 in case of Arg28 and G12 in case of Arg28′. Moreover, the two nucleotides G12 and G31 are base-paired to each other *via* a non-canonical *trans* sugar-edge/sugar-edge base pairing that leaves the Hoogsteen-edges accessible for hydrogen-bonding interactions with the arginine side chains (Figure 3B). This type of interaction has previously been highlighted as a Hoogsteen pseudo pair (36). The guanidinium group of Arg28 is also partially intercalated between the adenine rings of A15 and A30 and might form two cation-π interactions with theses nucleobases (Figure 3C). An additional sequence-specific hydrogen bond is established between Gln38 and G17 (Figure 3E). Due to the asymmetric nature of the interface, Arg28′ and Gln38′ from the second protomer neither participate in cation-π interactions (Arg28′) nor in guanine recognition (Gln38′).

As shown in Figure 3F, the flipped-out nucleobase U16 is stabilized by a π–π-stacking interaction with the side chain of Tyr42. The equivalent interaction of Tyr42′ appears to be more elaborate, involving not only the flipped G8, which stacks directly with Tyr42′, but also an additional layer of π-π stacking between G8 and the likewise flipped A33 (Figure 3D). In addition, the ϵ-amino group of Lys46′ is located within hydrogen-bonding distance of both G8 and G33. However, it was not possible to accurately determine the exact orientation of the side chain due to a lack of well-defined electron density. This indicates that the hydrogen bond is only formed in some molecules in the crystals and that, overall, this hydrogen bond does not contribute significantly to the free binding energy of the complex formation.

### Arg28 and Tyr42 represent major RNA aptamer-binding determinants in TetR

**Figure 4.**
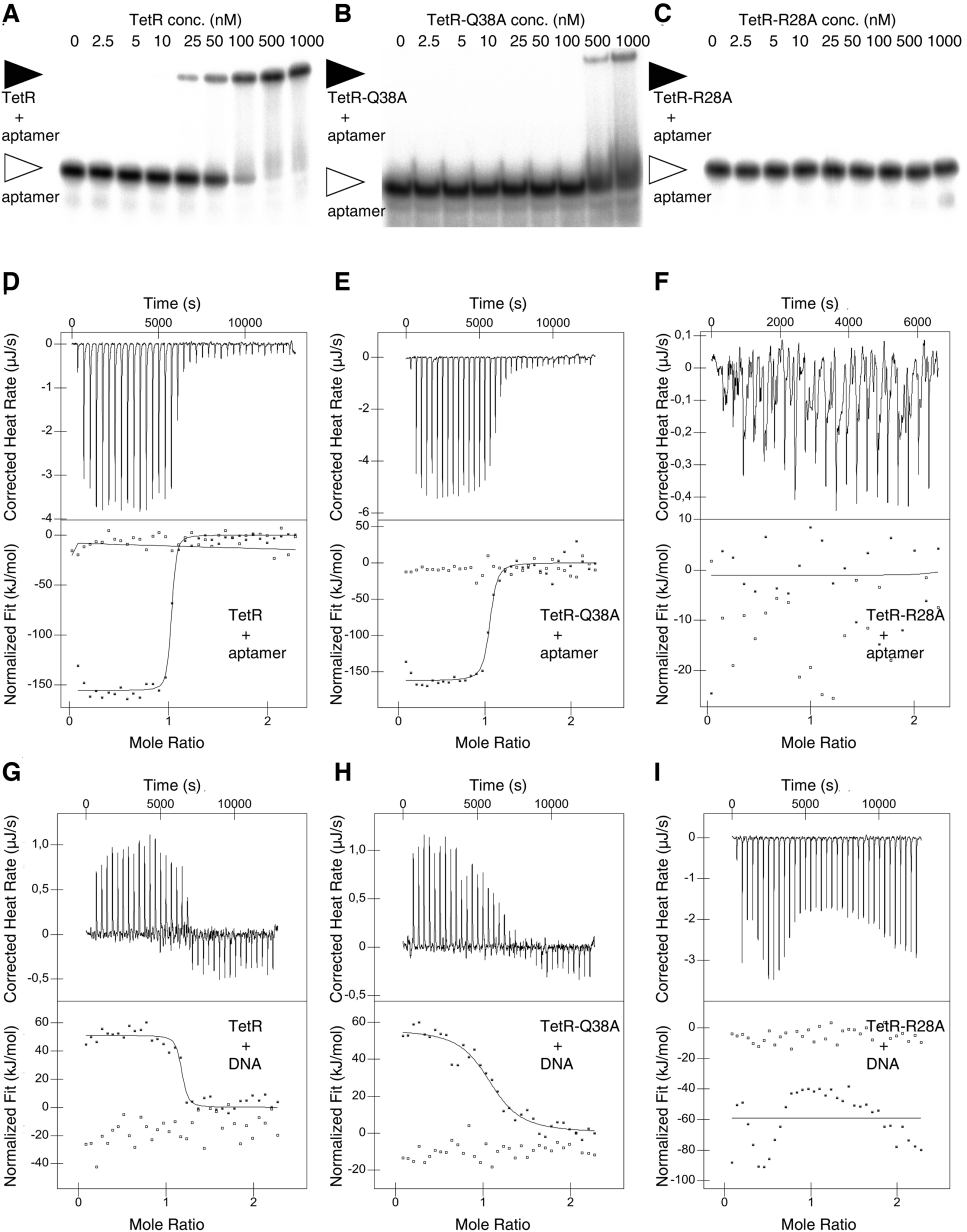
Analysis of the interaction of TetR and TetR mutant variants TetR-Q38A and TetR-R28A with either the RNA aptamer or *tetO* operator-encoding DNA. (**A**) EMSA showing the interaction of the RNA aptamer with wild-type TetR, (**B**) with TetR-Q38A and (**C**) with TetR-R28A. (**D**) ITC measurements showing the titration of the RNA aptamer with wild-type TetR, (**E**) titration with TetR-Q38A and (**F**) titration with TetR-R28A. (**G**) ITC measurements showing the titration of double-stranded *tetO* operator DNA with wild-type TetR, (**H**) with TetR-Q38A and (**I**) with TetR-R28A.

The affinity of the RNA aptamer K1 for TetR was investigated *via* EMSA and ITC experiments (Figure 4). While the EMSA experiment yielded a *K*_D_ of 80 nM, a *K*_D_ of 5.6 nM was recorded *via* ITC (Figure 4D, Supplementary Figure S4, Table 2). The ITC experiment also confirmed the 1-to-1 stoichiometry of the complex with one TetR dimer binding to one aptamer molecule (Table 3). The ITC measurements revealed that TetR–RNA aptamer complex formation is enthalpy-driven (Δ*H* = –156.0 kJ mol^-1^) and that complex formation coincides with a large entropy reduction (*T*Δ*S* = –108.8 kJ mol^−1^, Table 3).

**Table 2.** RNA and DNA-binding results

	RNA aptamer			*tetO* dsDNA	
	SEC	EMSA	ITC	SEC	ITC
Construct	Complex formation	*K*_d_ [nM]	*K*_d_ [nM]	Complex formation	*K*_d_ [nM]
TetR	Yes	80	5.6	yes	51.1
TetR–R28A	No	n.b.^a^	n. b.	no	n. b.
TetR–Q38A	Yes	>500^b^	26.2	yes	432.7
TetR–Y42A	Yes	n.b.	13.9 × 10^3^	no	n.b.
TetR–R28AY42A	No	n.b.	–^c^	no	–

^a^No binding detectable.

^b^Accurate determination of *K*_d_ not possible with the reported experimental setup.

^c^Not determined.

**Table 3.** Affinities and thermodynamic parameters of the TetR–RNA aptamer and TetR–DNA complexes

	*K*_d_ (nM)	*n* ^a^	Δ*H* (kJ mol^−1^)	Δ*S* (J mol^−1^ K^−1^)	–*T*Δ*S* (kJ mol^−1^)	Δ*G* (kJ mol^−1^)
TetR–RNA aptamer K1 complex formation						
TetR	5.6	0.96	−156.0	−365.2	108.8	−47.2
TetR–Q38A	26.2	1.10	−152.8	−367.2	109.4	−43.4
TetR–Y42A	13.9 × 10^3^	1^b^	−74.6	−157.1	46.8	−27.8
TetR–DNA *tetO* operator complex formation						
TetR	51.1	1.17	57.7	336.4	−100.2	−42.5
TetR–Q38A	432.7	1.11	53.4	300.9	−89.7	−36.3

^a^ *N* = 1 signifies that one TetR dimer interacts with one RNA aptamer molecule or dsDNA fragment.

^b^ *N* = 1 was kept fixed during data evaluation.

Alanine-scanning mutagenesis of residues Arg28, Gln38 and Tyr42 was performed to investigate how individual amino acid side chains contribute to complex formation. Four different TetR mutant variants were produced, i.e. TetR–R28A, TetR–Q38A, TetR–Y42A and TetR–R28A–Q38A, and their ability to bind to the RNA aptamer was first investigated using EMSA and SEC (Table 2). Complex formation was completely abolished in variants TetR–R28A, TetR–Y42A and TetR–R28A–Q38A, whereas weak aptamer binding could be detected with variant TetR–Q38A when probed in an EMSA experiment (Figure 4A–C, Supplementary Figure S4A).

Almost identical behaviors were observed in a SEC assay (Supplementary Figures S5A, B and S6A–D, Supplementary Table S3). There was no detectable complex formation for variants TetR-R28A and TetR–R28A–Q38A, whereas the chromatograms of the variants TetR–Y42A and TetR–Q38A suggested a partial complex formation, only. The amount of protein-bound aptamer was smaller in variant TetR–Y42A, hinting that the aptamer-binding affinity of this variant is lower than that of TetR–Q38A (Supplementary Figure S5B).

To better quantify the changes in binding affinity, a series of ITC experiments were performed (Figure 4D–F, Supplementary Figure S4B, Table 3). The absence of any binding heat during the titration of variant TetR–R28A confirmed that aptamer binding is abolished in this variant. When taking into account the signal detection limits of the ITC experiment, it can be estimated that the affinity of TetR–R28A for the aptamer must be less than 1 mM. This amounts to a >180 000-fold reduction in binding affinity in comparison to the wild-type protein. The binding affinity is also considerably reduced in variant TetR–Y42A (*K*_D_ = 13.9 μM, 2500-fold reduction), while only moderately reduced in variant TetR–Q38A (*K*_D_ = 26.2 nM, 5-fold reduction). The behavior of variant TetR–Q38A is in line with the observation that only one of the two Gln38 residues in dimeric TetR participates in aptamer binding, whereas in the case of residues Arg28 and Tyr42, both arginine and tyrosine residues are in contact with the aptamer. In sum, these results show that Arg28 and Tyr42 represent the major specific binding determinants in the TetR–RNA aptamer complex.

### RNA aptamer binding versus operator DNA binding to TetR

Binding of TetR to the *tetO* operator DNA and associated structural rearrangements in TetR have been extensively characterized in the past. Crystal structures of the TetR–DNA complex (PDB entry 1QPI, (13,37)), ligand-free TetR (1A6I, (38); 2NS7, (26)) and of tc-bound TetR (2TCT, (39)) are available along with extensive mutagenesis data investigating the contribution of individual TetR amino acids to DNA binding (40). Inspection of these data shows that the TetR–RNA aptamer complex shares considerable parallels with the TetR–DNA complex.

**Figure 5.**
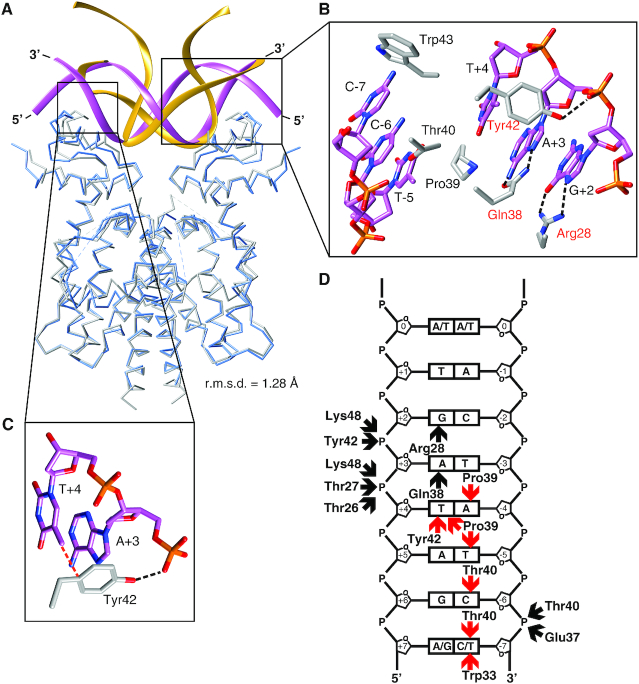
Comparison between the TetR–RNA and the TetR–DNA interface. (**A**) Superposition of the TetR-RNA aptamer (in blue and yellow) and the TetR–DNA complex (PDB entry: 1QPI) (gray and purple). A TetR–DNA interaction half-site is framed. The overall conformation of TetR is highly similar in both complexes (r.m.s.d. = 1.28 Å, calculated for 356 Cα atom pairs). (**B**) Detailed view of the TetR–DNA interaction half-site. TetR side chains directly interacting with DNA nucleotides are shown in a stick representation. Nucleotides are labeled according to (13). Red labels denote those amino acids of TetR that participate in interactions with both DNA and RNA aptamer. (**C**) Interaction between Tyr42, A+3 and T+4. In panels B and C, dotted black lines indicate hydrogen bonds and dotted red lines indicate van der Waals interactions. (**D**) Schematic representation of the interactions between TetR and one half-site of the operator DNA (calculated with Nucplot (53), see also Supplementary Figure S7A). In all panels, dotted black lines or black arrows indicate hydrogen bonds and dotted red lines or red arrows indicate van der Waals contacts.

In the TetR–DNA complex, TetR binds to the dsDNA segment from one side, and its two NBDs bind to two consecutive major groove segments that are separated by a minor groove segment (Figure 5A). The contact surface area between TetR and DNA extends over 1120 Å^2^ and, because of the palindromic nature of the bound dsDNA and dimeric nature of TetR, is identical for the two half-sites (560 Å^2^, each half site). Overall, the contact surface area of the TetR–DNA complex is ∼30% larger than that of the TetR–RNA aptamer complex (850 Å^2^, see above).

When comparing the overall conformation of DNA-bound TetR with that of RNA-bound TetR, it becomes apparent that the overall conformation of TetR is nearly identical in the two complexes (Figure 5A). Thus, superimposing the TetR-RNA aptamer complex onto various TetR structures using only the coordinates of the EBDs demonstrates that the resulting orientations of the NBDs in the TetR-RNA aptamer complex closely resemble the orientation of the NBDs in DNA-bound TetR (Table 4). They also resemble those observed in TetR in complex with the peptide TAP1, a peptide that has been shown to inhibit induction of TetR by tc (Table 4) (27). At the same time, the orientation of the NBDs differs significantly from those previously observed in structures of TetR in complex with the effector tc and the peptide TIP1. The latter peptide has been shown to be able to mimic the function of tc (Table 4) (26). These comparisons clearly show that of the two known and well characterized conformations of TetR, i.e. the DNA-binding-competent and the tc-induced conformation, TetR adopts the former in the TetR–RNA aptamer complex.

**Table 4.** Structural comparison of various TetR structures

	TetR–aptamer complex				
			Inter-residue distances between identical residues (Cα-positions) in the superimposed complexes [Å]^c^		
	r.m.s.d. EBDs [Å]^a^	r.m.s.d. NBDs [Å]^b^	Residue 55 on helix H4	Residue 12 on helix H1	Residue 42 on helix H3
TetR–TAP1 complex (PDB entry: 3ZQF, (37))	1.07	1.01	0.37	0.70	1.52
TetR–DNA complex (1QPI)	1.03	1.97	0.48	0.71	2.16
Tc-bound TetR (2TCT)	1.48	2.55	2.09	2.53	2.76
TetR–TIP complex (2NS8)	1.27	2.91	1.49	2.46	3.16

^a^R.m.s.d. values obtained upon superposition of the two EBDs, only. Please see the Materials and Methods section for details.

^b^Values obtained for the comparison of the orientation of the NBDs between the different complexes after the coordinates of the EBDs have been superimposed.

^c^Distances occur twice, and average values are reported.

TetR assumes an identical conformation for DNA and RNA aptamer binding, perhaps because this conformation has been specifically selected for during the SELEX procedure. This appears likely, since the DNA-binding-competent conformation largely coincides with the preferred conformation of ligand-free TetR in solution (26).

### RNA and DNA binding is accomplished by identical binding determinants

Inspection of the TetR–DNA complex crystal structure shows that a total of 22 residues from the TetR NBDs are located within 3.7 Å of any DNA atoms (13). Because of the 2-fold symmetry of the complex, each TetR chain contributes the same 11 residues to the interaction. Thus, a larger number of TetR residues participate in DNA than in aptamer binding (22 *versus* 13 residues, see above). In particular, the side chains of residues Pro39, Thr40, Trp43, Gln38, Tyr42 and Arg28 directly contact atoms from DNA nucleobases (Figure 5B) (13,41). However, whereas Pro39, Thr40, Tyr42 and Trp43 participate only in van der Waals interactions with atoms from nucleobases, the side chains of Gln38 and Arg28 are the sole side chains that participate in direct hydrogen-bonding interactions with nucleobases, specifically with the bases guanine and adenine located at positions +2 and +3, respectively, of the 15 bp-long *tet*-operator (Figure 5D; Supplementary Figure S7) (13). The side-chain of Tyr42 participates both in a van der Waals interaction with the thymine base at position +4 and in a hydrogen-bonding interaction with the 5′-phosphate group from the DNA backbone of the +2 nucleotide (Figure 5C) (13).

All residues important for TetR–RNA aptamer complex formation have been previously observed to be also relevant for the repressor activity of TetR. Thus, individual alanine substitutions of residues Thr27, Arg28, Leu41, Tyr42, Trp43 and His44 significantly reduced the TetR repressor activity in a transcription reporter assay (40). Moreover, residues Gln38, Pro39 and Thr40 were identified playing a major role in DNA recognition specificity (40). Thus, the combined list of functionally important residues in the TetR-DNA complex includes Arg28, Gln38 and Tyr42, which were identified as the major functional epitope in aptamer binding (see above).

The functional aptamer-binding epitope of TetR appears to be fully contained within the functional TetR DNA-binding epitope. To substantiate this hypothesis, we investigated the *tetO* DNA-binding behaviour of wild-type TetR and of mutants TetR–R28A, TetR–Y42A and TetR–Q38A using identical experiments as for aptamer binding (see above). TetR readily binds to the *tetO* dsDNA operator segment when investigated *via* SEC and ITC (Figure 4G-I, Supplementary Figures S4C, S5C/D and S6E–H, Supplementary Table S3). ITC measurements yielded a *K*_D_ value of 51.1 nM for the TetR–DNA complex (Table 2), which matches values reported in the literature (14 nM, (41)). A substitution of either Arg28 or Tyr42 by alanine completely abolished DNA binding, as observed in SEC and ITC experiments. In case of aptamer binding, substitution of Arg28 by alanine also abolished aptamer binding, and substitution of Tyr42 by alanine drastically reduced the aptamer-binding affinity (2500-fold, see above). Mutation of Gln38 against alanine led to an 8.5-fold reduction in DNA-binding affinity compared to a 4.5-fold reduction in aptamer binding (see above). These data show that Gln38, Tyr42 and Arg28 not only represent important binding determinants in both complexes, but that in addition, the variable extent to which these residues contribute to the binding affinity is also preserved in both the aptamer and DNA-binding complex.

Despite the similarities in binding affinities and in the contributions of selected residues to complex formation, a key difference between the TetR–RNA and TetR–DNA interaction lies in the thermodynamic parameters that drive complex formation (Table 3). Whereas TetR–aptamer complex formation is exothermic and enthalpy-driven, TetR binding to DNA is strongly endothermic with Δ*H* = 57.7 kJ mol^−1^. At the same time, TetR–DNA complex formation is only possible because of a considerable gain in entropy (*–T*Δ*S* = –100.2 kJ mol^−1^, entropy-driven reaction). However, the overall Δ*G* of –42.5 kJ mol^−1^ is very comparable to the aforementioned –47.2 kJ mol^−1^ of the TetR–RNA aptamer complex formation. These differences in the thermodynamic parameters also apply for the mutant variants (Table 3). For a number of TetR-like repressors, it has now been shown that DNA complex formation is entropy-driven (42–44). In cases where multiple repressor dimers bind cooperatively to an extended DNA operator segment, at least one of the binding steps appears to be entropy-driven (45,46).

## DISCUSSION

The TetR–RNA aptamer complex highlights the potential of the SELEX process for identifying high-affinity RNA aptamers that are able to out-compete natural interaction partners such as the binding of TetR to *tetO*. The affinity of the TetR-binding aptamer exceeds that of *tetO* operator DNA (5.6 versus 51.1 nM) when measured under identical conditions (Table 2). The structure of the TetR-aptamer complex shows that aptamer binding recruits amino acid side chains that are also involved in DNA binding, the most prominent being Arg28, Gln38 and Tyr42. These are responsible for conveying sequence-specificity *via* the formation of base-specific polar contacts. Despite utilizing similar contacts as the *tetO* operator DNA, the TetR-binding aptamer does not mimic the naturally occurring DNA fragment in its overall shape. RNA cannot adopt the canonical B-form DNA conformation. Instead, the aptamer adopts a sharply bent conformation in which the non-canonical *trans* sugar-edge/sugar-edge pairing between G12 and G31 acts as a hinge.

A similar behavior of a SELEX-derived aptamer has been observed in the crystal structure of the mammalian transcription factor NF-κB (p50_2_) in complex with an RNA aptamer (47). In this complex, the aptamer adopts a distorted A-form helical conformation to present a sequence of bases that mimic the interactions found in the p50-κB-DNA complex. The same was also observed for aptamers selected for the yeast TATA-binding protein TBP (48).

The TetR-binding aptamer was isolated from a pool of RNA molecules using specific selection criteria. Thus, the aptamer was selected for its ability to bind to TetR *in vitro* and to control TetR-regulated gene transcription *in vivo* through competing with DNA for TetR binding (10). There are a number of ways in which an aptamer with sufficient affinity for TetR could interfere with TetR binding to DNA. For instance, steric hindrance produced by a partial overlap of the binding sites could render a simultaneous binding of DNA and aptamer impossible. Alternatively, the aptamer could tap into the allosteric mechanism that is triggered in TetR upon binding of small effector molecules, such as tc (14). This tc-induced mechanism allosterically alters the distance between the NBDs of TetR, which also abolishes DNA binding.

Of these two possibilities, we observe that TetR-RNA aptamer binding almost perfectly mimics DNA binding of TetR. Instead of generating a mere steric overlap between binding sites, DNA and aptamer bind to identical structural binding epitopes on the TetR surface. This also extends to the functional epitope, i.e. identical residues contribute most free energy to complex formation with both DNA and aptamer (49). This suggests that the TetR protein encodes for a preferred mechanism for binding to nucleic acids that is independent of the nature of the nucleic acid molecule.

The thermodynamic characteristics of the complex formation reactions differ greatly between RNA and DNA binding. They may indicate that the unbound RNA aptamer displays a greater flexibility in solution than is the case for unbound operator DNA. TetR binding to *tetO* operator DNA is an entropy-driven process, whereas binding of TetR to the RNA aptamer is enthalpy-driven. Since the binding partner TetR is the same in both processes, the entropy versus enthalpy differences must be linked to either distinct properties of the free nucleotides and/or of the complexes. In the TetR-DNA complex, the operator DNA is slightly distorted in comparison to canonical B-form DNA (13). This distortion is likely the reason for the positive Δ*H* value of the TetR–DNA complex formation. At the same time, the rather stiff nature of an unbound short *B*-form DNA molecule reduces the entropy loss upon complex formation. Hence, the entropy gain produced by expelling water molecules from the interaction surface is able to overcome the positive Δ*H* and induce TetR-DNA complex formation.

When considering that the interfaces in the TetR–DNA and TetR–RNA complexes are of similar sizes, it can be assumed that the entropy gain obtained by the expulsion of water molecules from the interface area is similar in the two complexes. However, the overall entropy change is negative in the TetR–RNA aptamer complex formation and positive in TetR–DNA. Since TetR adopts an identical conformation in both complexes, the Δ*S* sign switch between both reactions should primarily originate from a substantially higher flexibility of unbound RNA aptamer in comparison to unbound DNA. This would explain a greater loss of conformational entropy during complex formation in case of the RNA aptamer.

TetR adopts the same overall conformation when bound to the TetR-binding aptamer as when bound to operator DNA. This conformation has been described in the literature as the DNA-binding-competent conformation of TetR (14). In the two-state allosteric model of TetR function, this conformation is in equilibrium with the so-called effector-induced conformation of TetR in which tc binding to the EBDs changes the separation of the NBDs and abolishes DNA binding. Surprisingly, binding of tc to TetR also prevents binding of TetR to the aptamer despite the fact that the TetR-binding aptamer identification procedure did not include any such selection pressure (10). This suggests on one hand that the structure of the aptamer in the complex is unable to accommodate any changes in the separation of the NBDs of TetR induced upon tc binding. On the other hand, what could be interpreted as a random emergence of tc-inducibility might in fact be a necessity that is an immediate consequence of the fact that TetR is able to sample only two distinct conformations. Once the DNA-binding-competent conformation of TetR has been selected as competent for aptamer binding, the tc-induced conformation of TetR is automatically incompatible with aptamer binding.

The nature of the allosteric mechanism of TetR remains controversial. Recently, it has been proposed that allostery in TetR is not, as initially suggested, ruled by a two-state allosteric model but rather by a ligand-induced folding mechanism that closely resembles the population shift model of allostery (50,51). According to this model, tc-free TetR samples multiple conformations, and among these, one is able to bind to DNA. Tc binding to TetR then causes the folding of TetR into a defined conformational state unable to interact with DNA anymore (50,51). While such a population shift model might best explain the behavior of some of the reverse TetR mutants, the present study strongly supports the validity of the classical two-state model for wild-type TetR (50,52). Although not biased for by the selection protocol, we observe that TetR binds to the RNA aptamer in exactly the same conformation as it binds to DNA. At the same time, its aptamer-binding affinity is modulated by the same tc-induced molecular switching mechanism that causes TetR to toggle between a DNA-binding-competent and non-binding conformation.

The two-state-only model is not only fully consistent with previous structural data on TetR, i.e. on TetR by itself, the TetR-tc and the TetR–DNA complex, but also in line with multiple structures of TetR in complex with synthetic peptides (14,27). As for the artificial TetR-binding aptamers, all these peptides cause TetR conformations that fall into either of the two conformations that characterize the two-state allosteric model of TetR (27). Thus, the current study presents new data that strongly support the validity of the ‘old’ two-state-model of allostery in TetR, which might in fact apply for all members of the TetR family.

One of the many interesting aspects of the TetR aptamer system is the fact that the tc-triggered allosteric mechanism remains in full use. This introduces an additional level of control that has been extremely advantageous for the more complex applications of the TetR-binding aptamer system. To our knowledge, it is currently the only protein-responsive mRNA switch where RNA binding can be reversibly controlled by a small molecule. The switch has been proven to be effective not only for the transcriptional control but also for regulation of translation, pre-mRNA splicing and miRNA processing (Figure 1). The system is not only applicable in bacteria and lower eukaryotes but also represents a substantial extension for the toolbox of mammalian synthetic biology that is so far very limited in number of genetic modules able to construct complex genetic circuits for cell engineering or therapeutics.

## DATA AVAILABILITY

The coordinates of the structure of the TetR-RNA aptamer K1 and TetR–RNA aptamer K2 complex have been deposited with the Protein Data Bank (PDB) with accession codes 6SY4 and 6SY6, respectively.

## Supplementary Material

gkaa083_Supplemental_File
