# Supplementary material for: The complex formed between a synthetic RNA aptamer and the transcription repressor TetR is a structural and functional twin of the operator DNA–TetR regulator complex
Source: Nucleic Acids Res. 2020 Feb 13;48(6):3366–78. doi: 10.1093/nar/gkaa083 (PMC7102968; doi:10.1093/nar/gkaa083)
Supplement: gkaa083_Supplemental_File [file gkaa083_supplemental_file.pdf]

Supplementary material

The complex formed between a synthetic RNA aptamer and the transcription repressor TetR is a structural and functional twin of the operator DNA-TetR regulator complex

Florian C. Grau, Jeannine Jaeger, Florian Groher, Beatrix Suess and Yves A. Muller

Supplementary Table S1. Nucleotide sequences of aptamers and DNA oligonucleotides

Aptamer K1	5’-GGCCGGAGAAUGUUAUGGCGCGAAAGCGCAGAGAAAACCGGUC-3’
Aptamer K2	5’-GGCGGAGAAUGUUAUGGCCUUCGGGCAGAGAAAACCGUC-3’
tetO dsDNA	strand I: 5’- CGCATTCTATCATTGATAGGATGCG -3’[a]<br>strand II: 5’- CGCATCCTATCAATGATAGAATGCG - 3’

[a] The underlined nucleotides form the palindromic half sites to which TetR binds.

Supplementary Table S2. Crystallographic data collection and refinement statistics of TetR in complex with the RNA aptamer K2

	**Complex TetR-K2**
**Protein data bank accession number**	6SY6
**Data collection**	
X-ray source	BESSY MX 14.2
Wavelength (Å)	0.9184
Space group	$P\ 3_121$
Cell dimensions	
a, b, c (Å)	96.56 96.56 119.97
α, β, γ (°)	90 90 120
Resolution range (Å)	16.1 - 2.9 (3.0 - 2.9)[a]
R_{meas} (%)	83.5 (579.9)
$CC_{1/2}$	0.975 (0.241)
CC^*	0.994 (0.623)
$I/\sigma I$	4.6 (0.5)
Completeness (%)	99.2 (100.0)
Redundancy	20.3 (21.4)
Wilson B-Factor ($Å^2$)	68.05
**Refinement**	
Resolution (Å)	16.1 - 2.9
No. reflections	14683 (1443)
$R_{\text{work}}/R_{\text{free}}$ (%)	25.0/28.7
CC_{work}	0.937 (0.417)
CC_{free}	0.920 (0.342)
No. atoms	
Macromolecules	3635
Ligands	-
Solvent	-
B-factors ($Å^2$)	
Mean	75.31
Macromolecules	75.31
Ligands	-
Solvent	-
R.m.s. deviations	
Bond lengths (Å)	0.005
Bond angles (°)	0.930
Ramachandran plot	
Favored (%)	98.28
Allowed (%)	0.0
Outliers (%)	0.0
Molprobity Clashscore	6.84

[a] Values referring to the highest-resolution shell are reported in parentheses.

Supplementary Table S3. Quantitative evaluation of the TetR-aptamer *versus* TetR-DNA complex formation rate *via* size exclusion chromatography

	Complex (V_E = 1.9 ml)[a]	TetR-apo (V_E = 2.0 ml)	Aptamer-apo (V_E = 2.2 ml)
TetR + aptamer	+++	-	+
R28A + aptamer	-	+	+++
Q38A + aptamer	++	-	++
Y42A + aptamer	+	-	++
	Complex (V_E = 1.7 ml)	TetR-apo (V_E = 2.0 ml)	*tetO*-apo (V_E = 1.9 ml, 2.2 ml)
TetR + *tetO*	+++	-	++
R28A + *tetO*	-	-	+++
Q38A + *tetO*	+		++
Y42A + *tetO*	-	-	+++

[a] V_E = Elution volume

Supplementary Figure S1

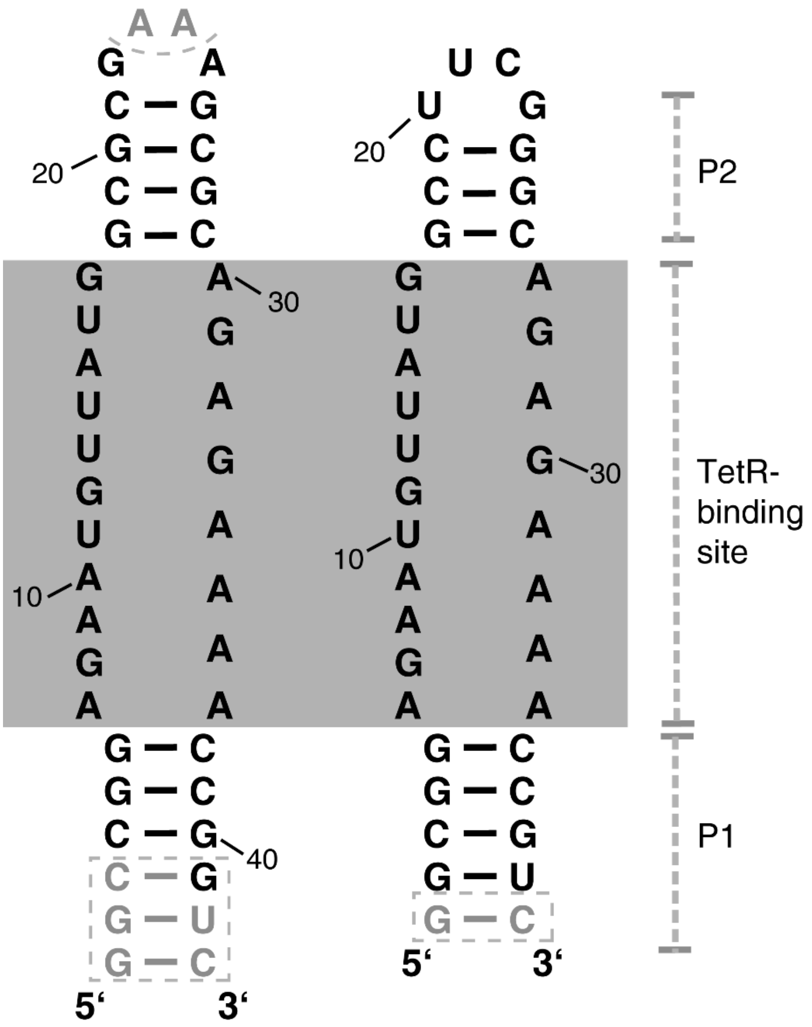

Supplementary Figure S1. Schematic representation of the TetR-binding aptamers K1 and K2. Nucleotides printed in black highlight those nucleotides that are visible in the crystal structure of the respective complex. Conversely, nucleotides printed in grey could not be resolved in the crystal structures (see also Figure 1 and Supplementary Figure S2). Canonical Watson-Crick base-pairings are represented as horizontal black lines. 5’ and 3’ ends and the two stems P1 and P2 are labelled.

Supplementary Figure S2

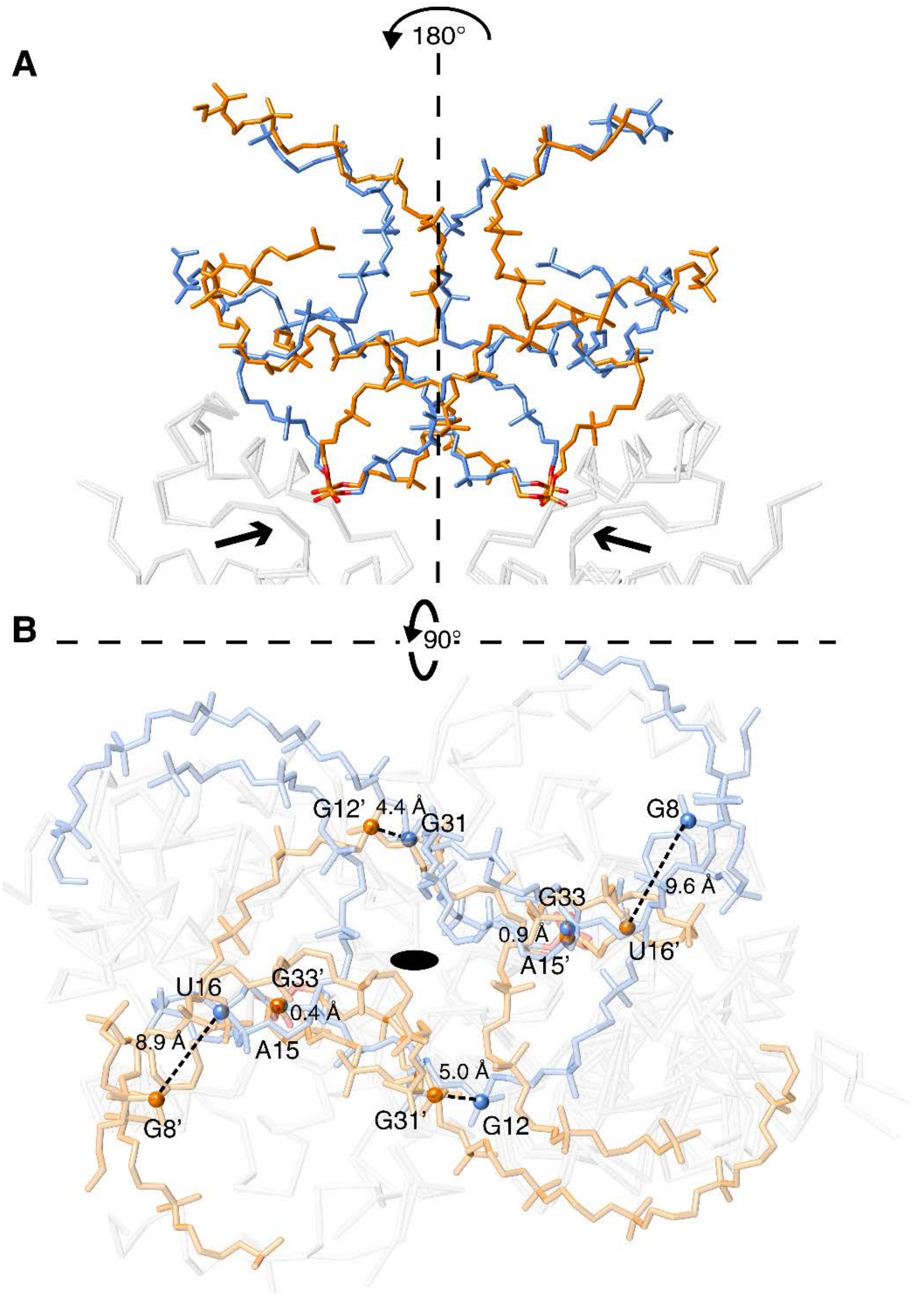

Supplementary Figure S2. Absence of a pseudo-symmetric dyad axis in the RNA aptamer structure. (**A**) Superposition of the NBDs of the TetR-RNA aptamer complex with the NBDs of a copy of the complex that, beforehand, has been rotated by 180° along the dyad axis that characterizes the TetR homodimer (shown as a vertical dotted line). Following this operation, the two aptamer copies deviate considerably from each other. Only the phosphate moieties of nucleobases A15 and G33 are congruent with their counterparts G33’ and A15’, respectively. (**B**) Superposition as in (A) but viewed from the top and along the TetR dyad axis (black ellipse). The backbone phosphor atoms from selected nucleotides involved in sequence-specific interactions with TetR are shown as spheres and the distances between geometrically equivalent pairs are highlighted with dotted lines.

Supplementary Figure S3

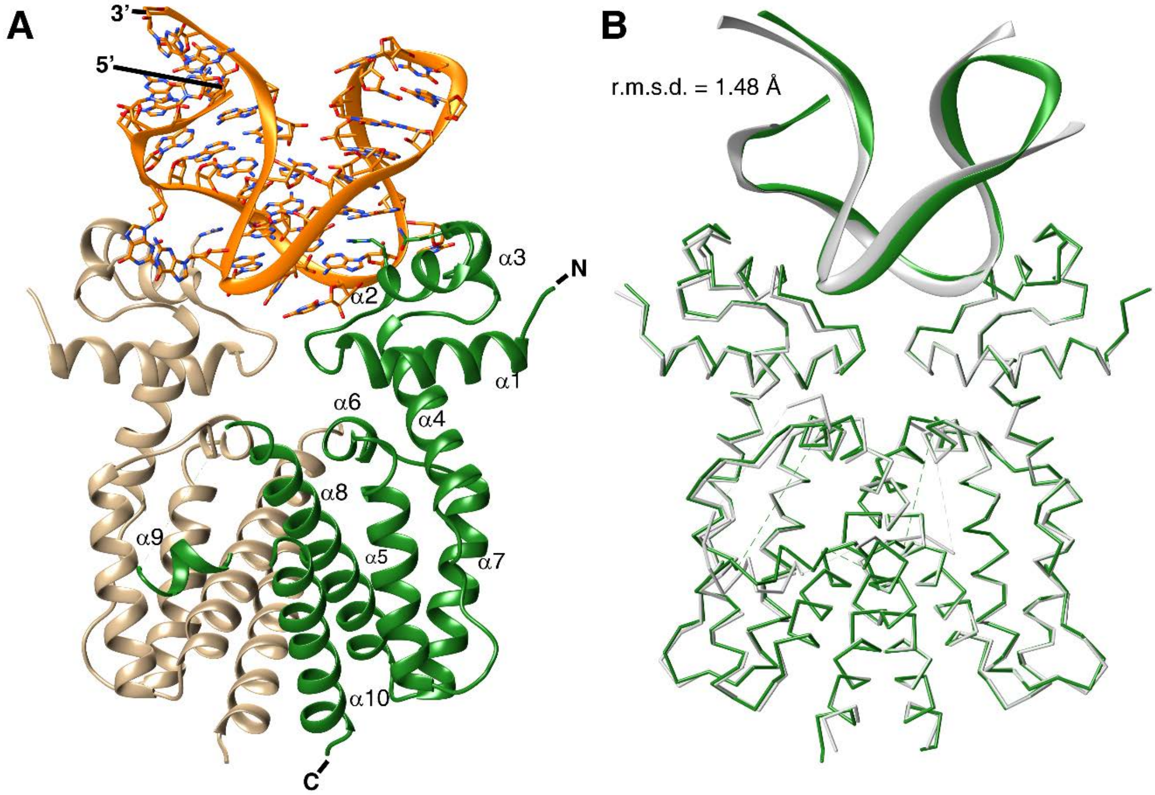

Supplementary Figure S3. Structure of the TetR-RNA aptamer K2 complex. (**A**) TetR homodimer in complex with the TetR-binding aptamer K2 shown in a cartoon representation with the TetR monomers colored in green and beige. All RNA bases, as well as amino acid side chains, which interact directly with the aptamer, are shown as sticks. The α-helices are labelled from α1 to α10 in one TetR monomer. (**B**) Superposition of the TetR-RNA aptamer K1 (gray) and TetR-RNA aptamer K2 (green) complexes. The complexes deviate by an r.m.s.d. value of 1.48 Å (calculated using 357 Cα and 32 phosphor atom pairs).

Supplementary Figure S4

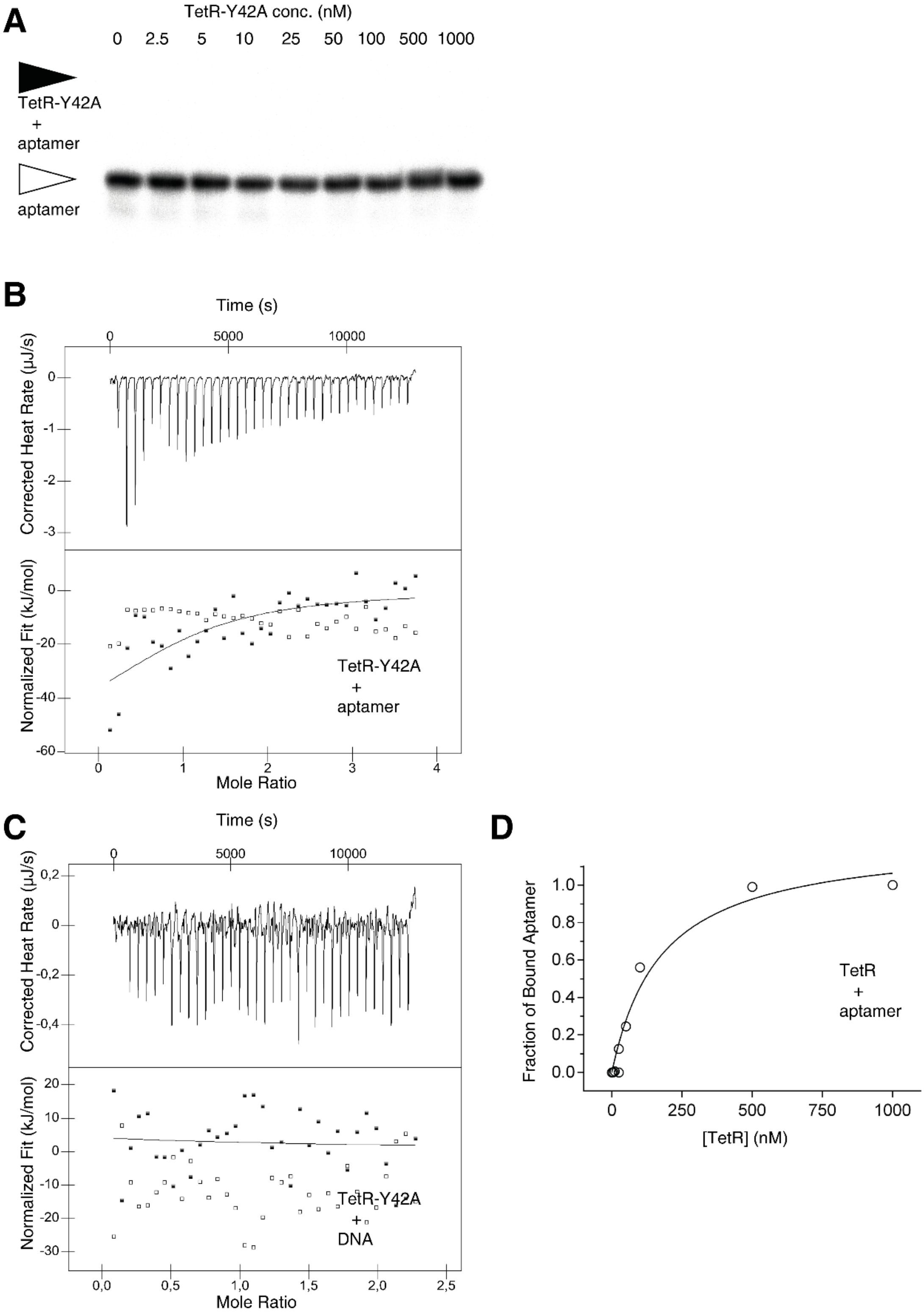

Supplementary Figure S4. Quantitative analysis of the interaction of wild-type TetR and TetR-Y42A with the TetR-binding aptamer compared to the binding of TetR-Y42A to a 25 bp-long, double-stranded DNA fragment containing the *tetO* operator sequence. (**A**) Electrophoretic mobility shift assay investigating the interaction of TetR-Y42A with the TetR-binding aptamer. (**B**) ITC experiment showing the titration of TetR-Y42A with the aptamer. (**C**) ITC measurement with TetR-Y42A titrated to double-stranded *tetO* operator DNA. (**D**) EMSA-derived binding isotherm of wild-type TetR and aptamer.

Supplementary Figure S5

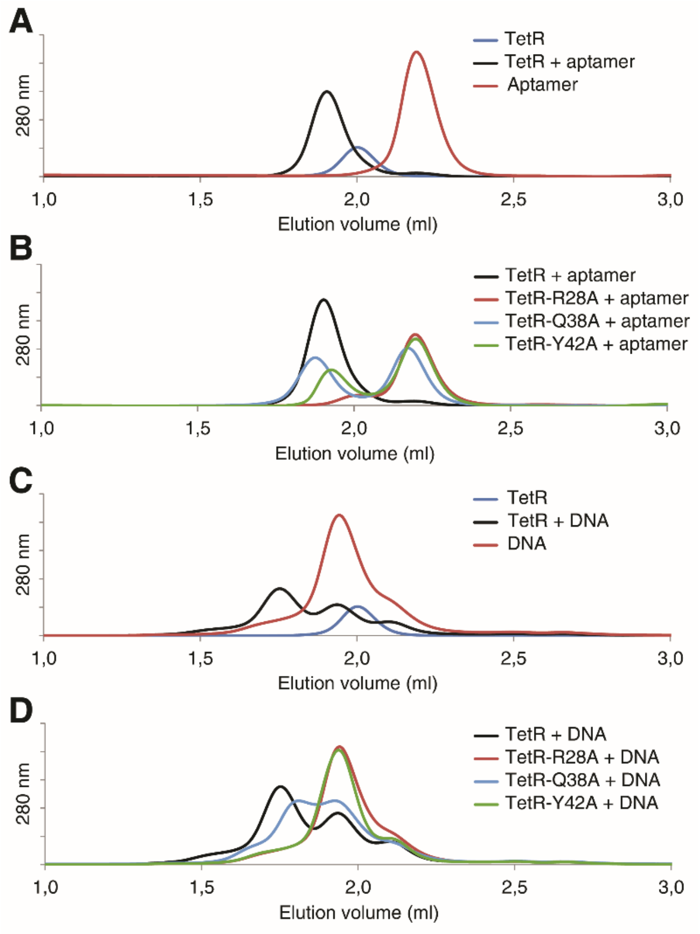

Supplementary Figure S5. Qualitative analysis of TetR-RNA aptamer and TetR-DNA complex formation. (**A**) Superposition of SEC runs of wild-type dimeric TetR (in blue), TetR-binding RNA aptamer (red) and the TetR-RNA aptamer complex (black). The chromatogram demonstrates the TetR-RNA aptamer complex formation. (**B**) Superposition of SEC runs of wild-type TetR (black, as shown in panel A), TetR-R28A (red), TetR-Q38A (blue) and TetR-Y42A (green), each upon addition of the TetR-binding RNA aptamer. Variants TetR-Q38A and TetR-Y42A display an incomplete complex formation whereas no complex formation is observed with TetR-R28A. (**C**) Superposition of SEC runs of wild-type, dimeric TetR (blue), 25 bp, double-stranded *tetO* operator DNA (red) and of TetR plus *tetO* operator DNA (black). The chromatogram demonstrates the formation of a TetR-*tetO* complex. (**D**) Superposition of SEC chromatograms of *tetO* operator DNA plus wild-type TetR (black, as seen in panel C), TetR-R28A (red), TetR-Q38A (blue) and TetR-Y42A (green). Whereas no DNA complex is formed with TetR variants TetR-R28A and TetR-Y42A, variant TetR-Q38A shows an incomplete and weakened complex formation.

Supplementary Figure S6

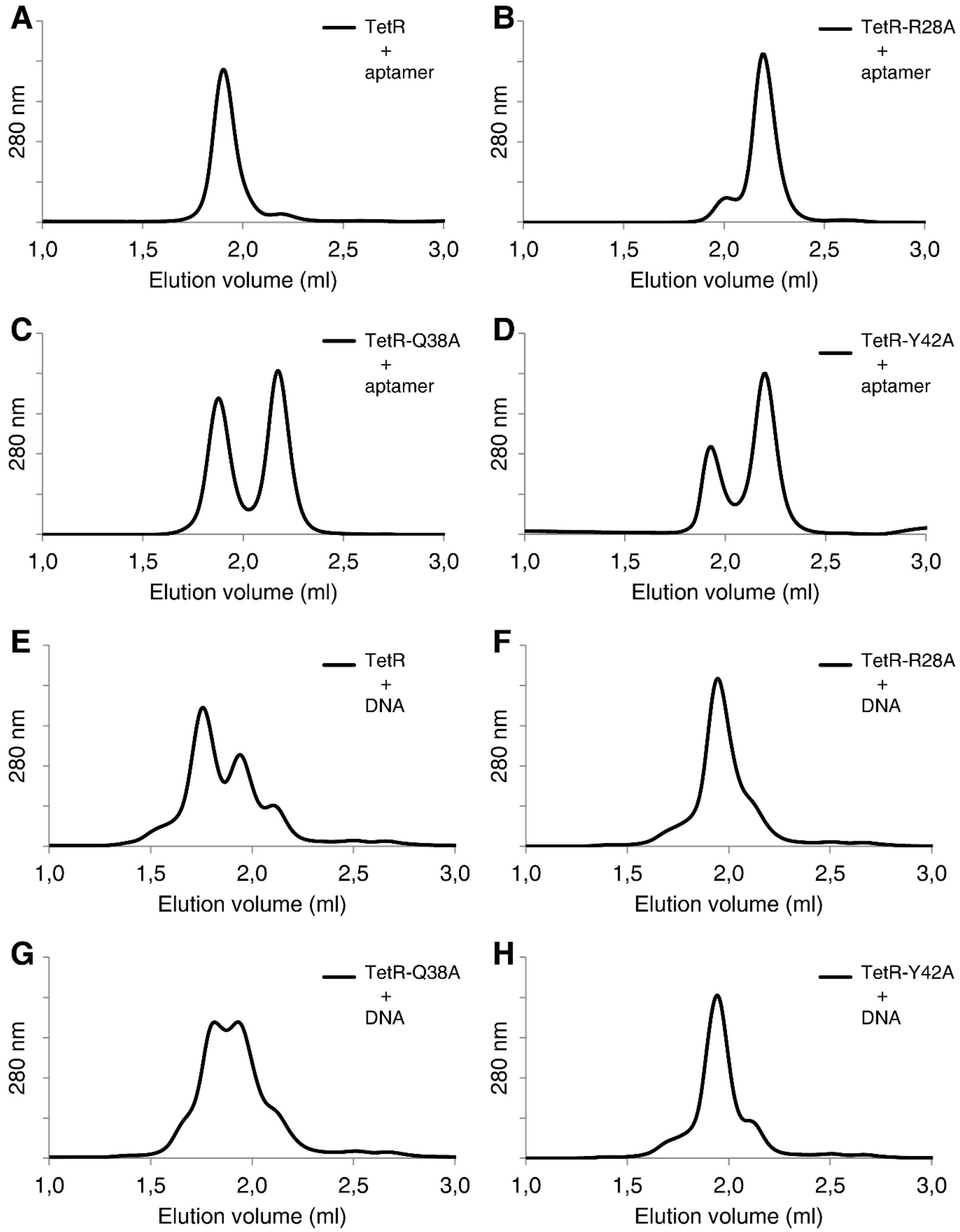

Supplementary Figure S6. Qualitative analysis of TetR-aptamer and TetR-DNA complex formation by SEC. Analytical SEC runs performed on a Superdex 200 column with equimolar amounts of dimeric TetR or mutant variants with either TetR-binding aptamer or double-stranded *tetO* operator DNA. SEC runs of (**A**) wild-type TetR and aptamer, (**B**) TetR-R28A and aptamer, (**C**) TetR-Q38A and aptamer, (**D**) TetR-Y42A and aptamer, (**E**) wild-type TetR and *tetO* operator DNA, (**F**) TetR-R28A and *tetO* operator DNA, (**G**) TetR-Q38A and *tetO* operator DNA, (**H**) TetR-Y42A and *tetO* operator DNA. A quantitative evaluation of the SEC data is provided in Supplementary Table S3.

Supplementary Figure S7

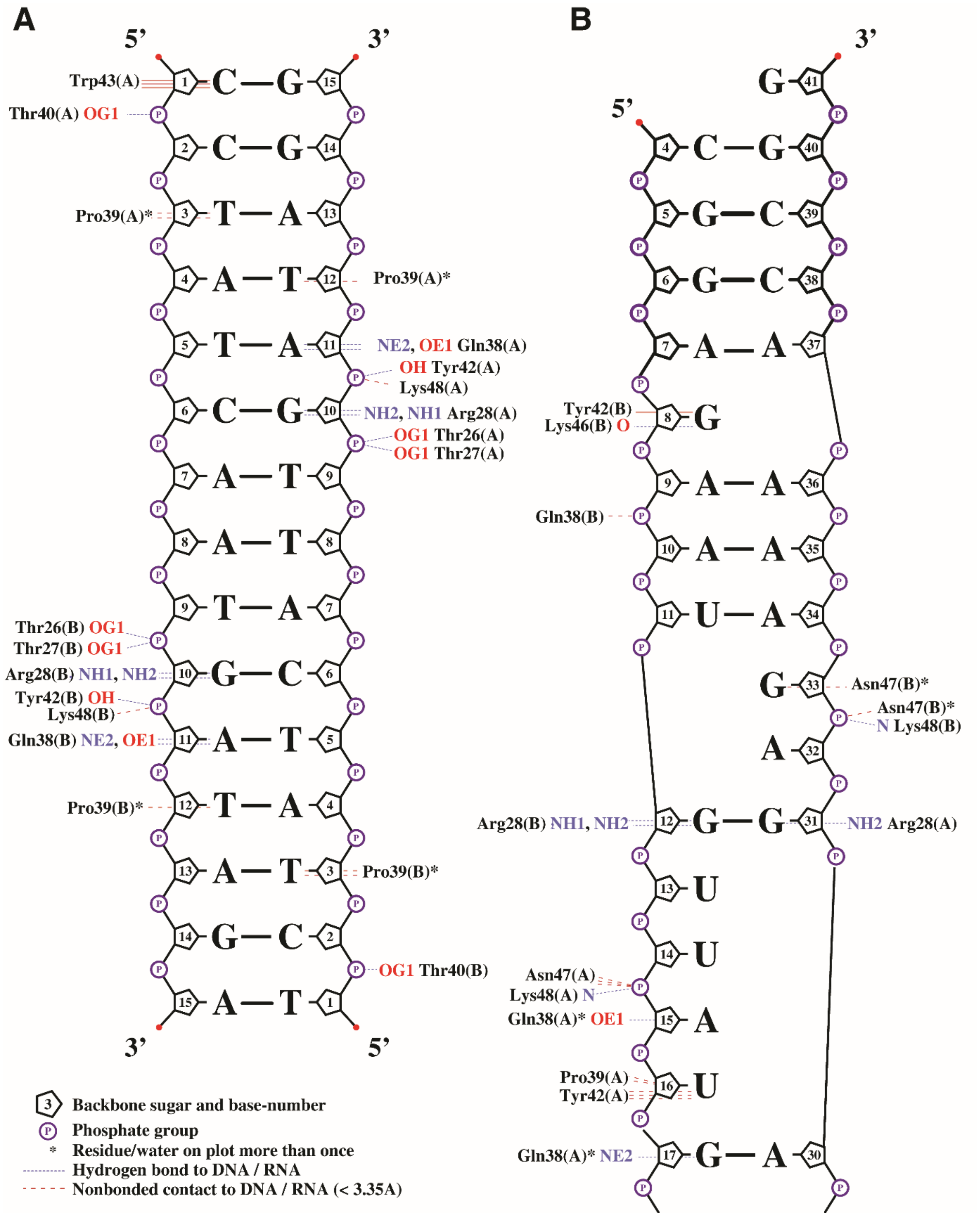

Supplementary Figure S7. Schematic representation of the TetR-DNA and TetR-RNA interfaces. Diagrams of the interfaces of (**A**) TetR and the 15bp *tetO* operator DNA and (**B**) TetR and the TetR-binding aptamer K1 generated with Nucplot (1).

Supplementary references

1. Luscombe, N.M., Laskowski, R.A. and Thornton, J.M. (1997) NUCPLOT: A Program to Generate Schematic Diagrams of Protein-Nucleic Acid Interactions. *Nucleic Acids Research*, **25**, 4940-4945.
